# How I do it. Foramen magnum meningiomas. Surgical tenets for a unique pathology of a transition zone

**DOI:** 10.1007/s00701-026-07006-y

**Published:** 2026-09-22

**Authors:** Sergio Corvino, Antonio Bernardo, Giuseppe Corazzelli, Giuseppe Catapano

**Affiliations:** 1 Department of Neurosurgery, “Ospedale del Mare”, 80147 Naples, Italy; 2 Department of Neurological Surgery, Weill Cornell Medicine/NewYork-Presbyterian Hospital, New York, NY USA; 3 Department of Neurosurgery, “La Sapienza” University of Rome, Rome, Italy; 4 Division of Neurosurgery, “Santa Maria delle Grazie” Hospital, Pozzuoli, 80078 Naples, Italy

**Keywords:** Foramen magnum, Meningiomas, Skull base, Brain tumors, Far lateral approach

## Abstract

**Background:**

Foramen magnum meningiomas (FMMs) represent significant neurosurgical challenge because of their intimate relationship with highly functional and vital neurovascular structures.

**Method:**

We discuss and illustrate technical nuances to resect type 3 FMMs with anterolateral dural attachment, causing compression and displacement of the medulla oblongata, accessory nerve and upper cervical spinal cord, with encasement of vertebral artery, through far lateral approach.

**Conclusion:**

Proper extradural preparation, early cerebrospinal fluid drainage, sectioning of dentate ligament, working between the nerve rootlets from caudal to rostral and from lateral to medial can enhance the safety and extent of resection.

**Supplementary Information:**

The online version contains supplementary material available at https://doi.org/10.1007/s00701-026-07006-y.

## Relevant surgical anatomy

**Fig. 1:**
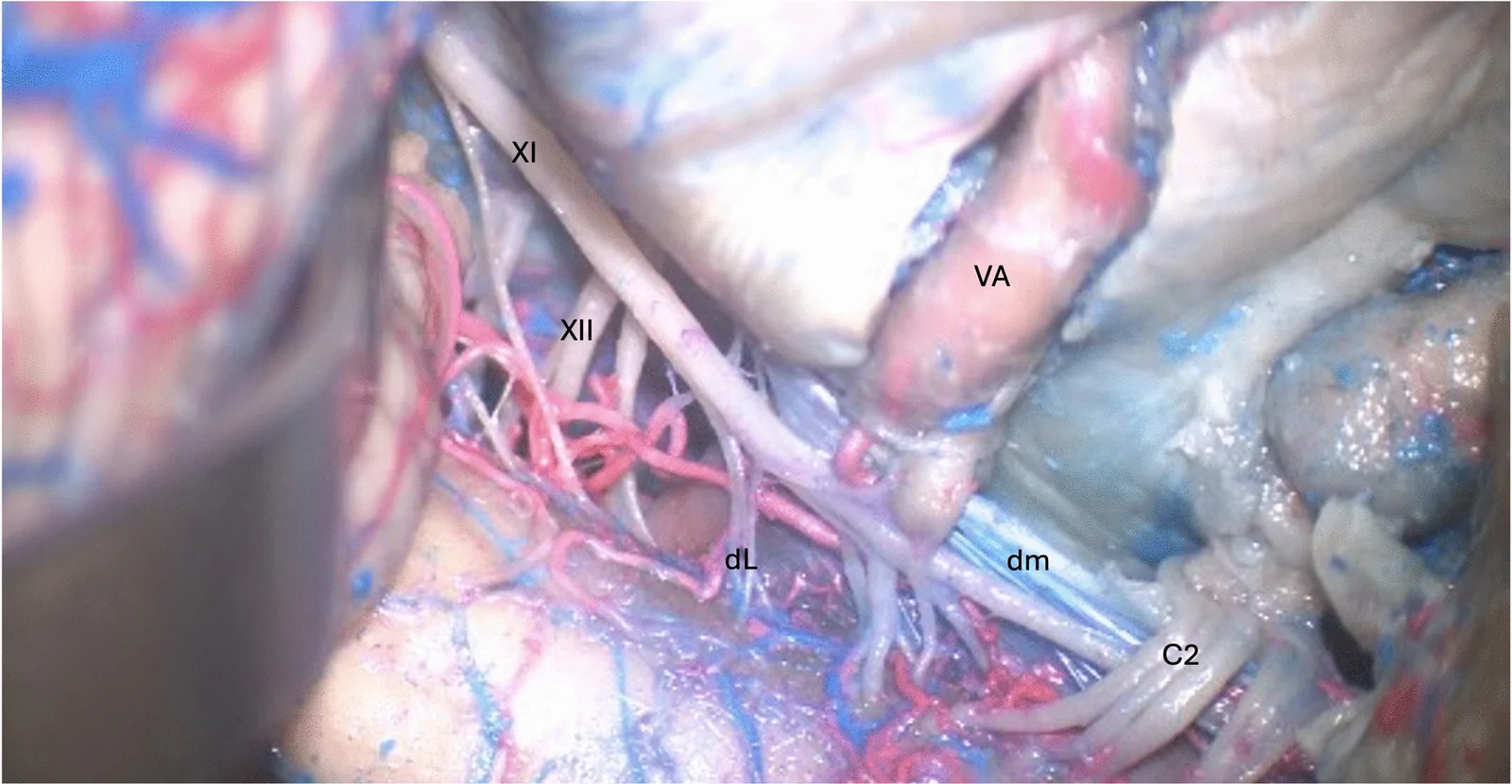
Microsurgical anatomy of the foramen magnum region in a cadaveric specimen. Right side. XI: accessory cranial nerve; XII: hypoglossal cranial nerve; VA: Vertebral artery; dm: dura mater; dL: dentate Ligament; C2: spinal cranial nerve root

Foramen magnum region represents the transition between the cranial cavity and the upper cervical spine and is characterized by a complex arrangement of osseous, neural, and vascular structures [3] (Fig. 1). It is bounded anteriorly by the clivus, laterally by the occipital condyles and jugular tubercles, and posteriorly by the squamous part of the occipital bone. The lower brainstem (medulla oblongata) continues caudally as the cervical spinal cord at this level, surrounded by the meninges and cerebrospinal fluid spaces. Critical neurovascular elements traverse this region, including the vertebral arteries (VA), which ascend through the transverse foramina of C1 and curve medially to enter the dura, and the lower cranial nerves (IX–XII), with particular surgical relevance of the hypoglossal nerve coursing through the hypoglossal canal and the accessory nerve rootlets ascending through the foramen magnum. The dentate ligaments provide lateral stabilization of the spinal cord and serve as important anatomical landmarks during surgical approaches.

## Description of the technique

In park bench position, a standard far lateral approach is performed including removal of the ipsilateral posterior arch of C1 and drilling of the jugular process while preserving the occipital condyle. The craniotomy begins from the foramen magnum lateral to the midline, continues medially up to the sigmoid sinus and jugular tubercle, and terminates at the contralateral aspect of the foramen magnum [2]. For further lateral and ventral exposure, the extradural drilling is extended to the lateral aspect of the foramen magnum at the jugular process. The dura is opened in a curvilinear fashion beginning in the midline from C2 to foramen magnum, then is turns over the exposed cerebellar hemisphere laterally toward the sigmoid sinus.

**Fig. 2:**
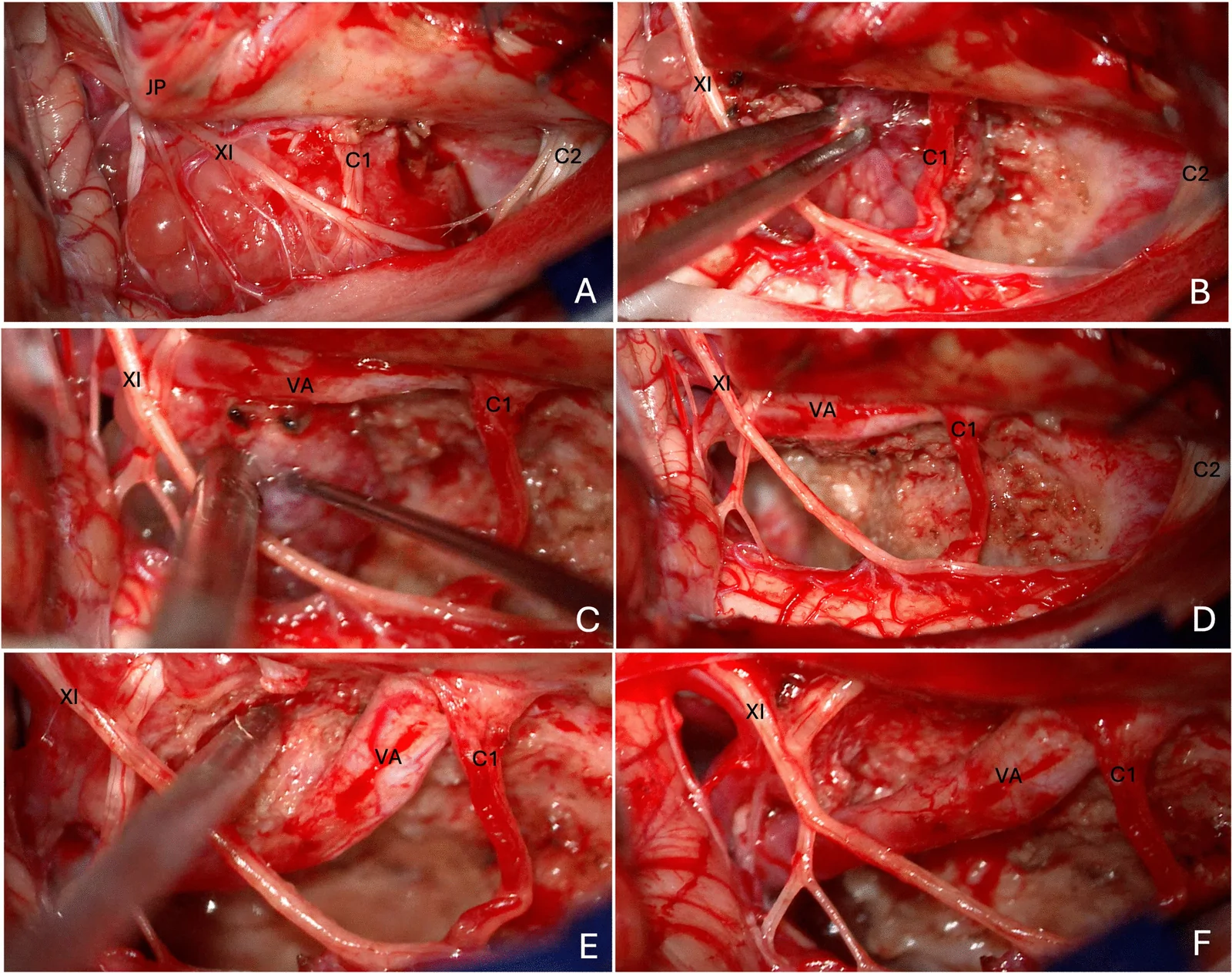
Intraoperative microsurgical tumor resection imagines. Right far lateral approach. Exposure of the lesion, with caudal extension up to C2 nerve, with dorsal displacement of the main trunk of accessory nerve (XI) and C1 nerve root (**A**); the most caudal portion of the tumor between C1 and C2 nerve roots (**B**), first, and the portion above C1 nerve root (**C-D**), then, are removed; thus, we addressed the most rostral part of the tumor, laterally to the vertebral artery (VA), that was resected (E); finally, we addressed the lesion around the VA, that was carefully isolated (**E**,**F**). (JP: Jugular Process; C1: first spinal cervical nerve root; C2: second spinal cervical nerve root; XI: accessory cranial nerve; VA: Vertebral artery)

After opening the dura mater, a type 3 FMM configuration [4] is observed, with the main trunk of the XI c.n. and the C1-C2 rootlets dorsally stratified over the tumor capsule (Fig. 2A). The opening of magnum cistern allows CSF drainage and cerebellum relaxation. Some authors [1] to prevent firing of the accessory nerve during surgery usually place small pieces of gelfoam soaked in preservative free 1% lidocaine on top of individual accessory nerve rootlets to achieve nerve paralysis. We don’t agree with this maneuver. By working constantly around it, the nerve could be unintentionally prolongedly stretched; therefore, instead of “silencing” the nerve, we prefer to receive constant feedback by the nerve during surgery.

The intracranial insertion of the dentate ligament is sectioned, allowing gentle medial mobilization of the medulla and spinal cord. This maneuver provides several surgical advantages: opens the route for a navigable corridor between spinal cord, brainstem, cranial nerves and the vertebral artery, increasing the safe working space for access to dural attachment for tumor devascularization, facilitation of intra-capsular debulking which leads to tumor capsule collapse, and increased exposure of medullary/spinal cord-tumor interface for subsequent dissection.

Tumor resection is then performed by working between the C1-C2 rootlets and the accessory nerve, avoiding their sacrifice, starting by coagulating the dural base between C1 and C2, followed by intracapsular tumor debulking with ultrasound aspiration and subsequent dissection of the capsule from the spinal cord medially and its subsequent removal (Fig. 2B). This sequence of maneuvers is repeated proceeding cranially, for the tumor component between C1 nerve root and the course of XI c.n. (Fig. 2B-D), and around the VA (Fig. 2E-F). A piecemeal resection proceeding from lateral to medial and from caudal to cranial, working in the space between the nerve roots, is advisable, by alternating bipolar electrocauterization of the dural implant, cutting with micro-scissors, tumor detachment from the dural base, and intracapsular debulking with ultrasound aspirator. Intra-capsular debulking allows the inward collapse of the tumor capsule, favoring the exposure of the tumor-brainstem/spinal cord interface, without the need for excessive medial mobilization of the nervous structures. In type 3 FMMs, lower cranial nerves are stretched dorsally to the superior pole of the tumor, therefore proceeding with the piecemeal resection from caudal to cranial direction allows for the progressive releasing of the involved nerves by working caudally and underneath without touching them.

### Illustrative case

A 60-year-old man was observed for 4-month history of progressive right-sided hearing loss.

**Fig. 3:**
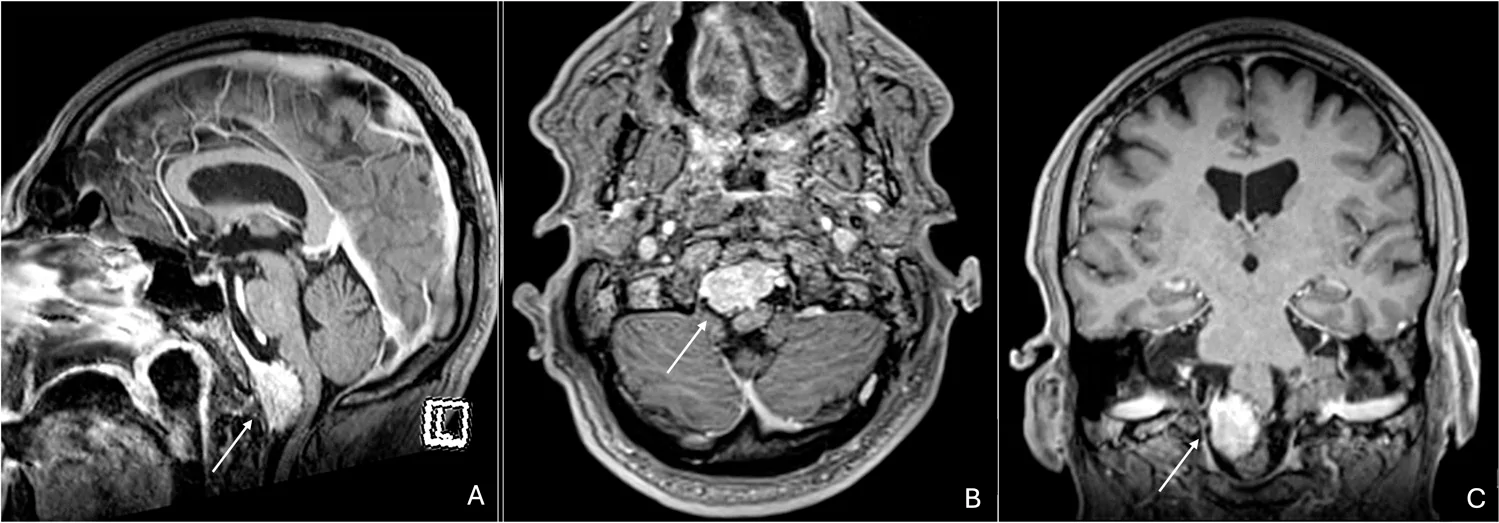
Preoperative post-contrast Magnetic Resonance Imaging (MRI) of the brain. Sagittal (**A**), axial (**B**) and coronal (**C**) sequences. Lesion (white arrow) with homogeneous contrast-enhancement extending from the intracranial surface of lower clivus through the foramen magnum to the vertebrae C1-C2, with anterior implant on the clival dura, partial encasement of the forth segment of right vertebral artery, exercising compression and displacement on the medullary-upper cervical spinal cord junction, consistent with a WHO grade 1 meningioma

Contrast-enhanced magnetic resonance imaging (MRI) of brain integrated by angiography sequences showed a type 3 FMM (Fig. 3).

Patient underwent a standard right far lateral approach. The operative procedure was conducted under intraoperative monitoring (IOM), including motor and somatosensory evoked potentials, as well as electromyographic monitoring of lower cranial nerves. A temporary pacemaker was also employed.

Early postoperative care was focused on neurological monitoring, airway protection, and assessment of lower cranial nerve function. Patients are routinely observed in the intensive care unit during the first postoperative night. Particular attention is paid to swallowing function, cough effectiveness, and signs of aspiration. The postoperative course was uneventful, and patient was discharged on postoperative day 5 without neurological deficit.

**Fig. 4:**
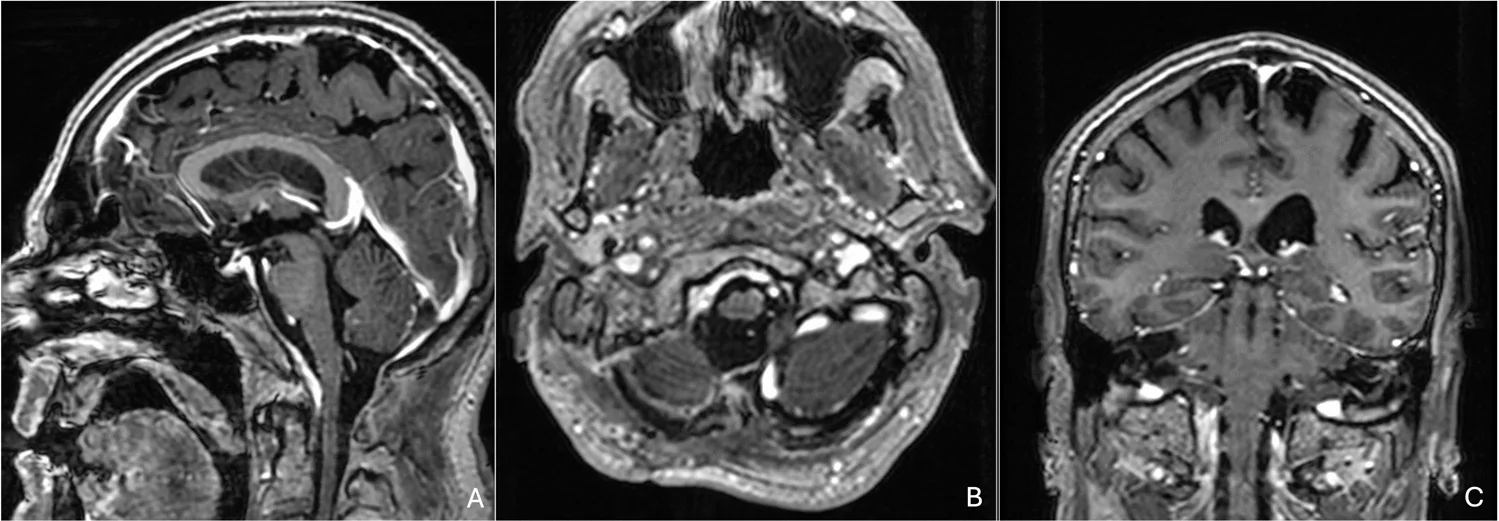
Postoperative post-contrast Magnetic Resonance Imaging (MRI) of the brain. Sagittal (**A**), axial (**B**) and coronal (**C**) sequences. No evidence of significant residual tumor and decompression of the medulla and cervical spinal cord

The post-contrast MRI of the brain at 3 months (Fig. 4) demonstrated no evidence of significant residual tumor and the resolution of the brainstem and spinal cord compression.

## Indications

In type 3 FMMs the paradigm of treatment is a symptoms-oriented surgery, prioritizing progressive neural decompression through piecemeal debulking and corridor-expanding maneuvers, while avoiding forced dissection in high-risk neurovascular interfaces.

## Limitations

The main limitation is not access, but the safety. The goal of surgery aiming gross-total resection (GTR) while preserving neurological function [5] in symptomatic and good performance status patients is not always achievable due to the tumor intimate relationship with several vital and highly functional neurovascular structures; it is reported in only 80% of cases and is achievable especially in small lesions and non-anterior locations [6]. The complexity of surgical treatment of FMMs is witnessed by the high overall mean complication and mortality rates, respectively of 17.2% and 16%, with the anterior tumor location, tumor invasiveness and extradural extension, recurrences with adhesion, VA encasement, arachnoid plane lost and tumor size representing the tumor-related risk factors of increased morbidity [6].

It is worth to remember that when subtotal resection is achieved in WHO Grade II FMMs with high values of Ki67-MIB1, it could be required adjuvant radiotherapy close to vulnerable neurovascular structures.

## How to avoid complications

The detailed knowledge of the microsurgical anatomy of the FM region, the meticulous interpretation of preoperative imaging, respect of the sequences of surgical steps, the assistance of IOM and ultrasound aspirator, can significantly improve the safety of the operative procedure.

## Specific information to give to the patient about surgery and potential risks

The goal of surgery is not necessarily gross total tumor resection, but to decompress brainstem, cranial nerves and spinal cord, to achieve symptoms relief and arrest neurological deficit progression. Risks of lower cranial nerves deficit, motor and somatosensitive deficits, must be illustrated to the patient.

## Ten key points

1. Foramen magnum meningiomas represent a formidable surgical challenge because of their intimate relationship with vital and highly functional neurovascular structures.
2. Goal of surgery: balance between extent of resection and preserve/restore arrest progression of neurological deficit.
3. Proper extradural preparation, including the drilling of the jugular process, improves anterolateral access.
4. Early cerebrospinal fluid drainage through subarachnoid cisterns opening increases safe working area.
5. Systematic identification of relevant neurovascular structures is mandatory.
6. Sectioning of the upper two triangular processes of dentate ligament expands the operative corridor.
7. Sacrifice of some rootlets of C1 nerve and/or of accessory nerves could be sometimes required.
8. Rather than “silencing” the accessory nerve, we prefer to receive constant feedback by it during surgery.
9. A piecemeal resection proceeding from lateral to medial and from caudal to cranial, facilitates safe tumor mobilization while minimizing neural traction.
10. These strategies allow maximization of tumor resection, reduction of recurrence risk and effective brainstem/spinal cord, cranial nerves decompression, ultimately leading to improved clinical outcome.

## Supplementary Information

Below is the link to the electronic supplementary material.

Supplementary file1 (MP4 417930 KB)

## Data Availability

No datasets were generated or analysed during the current study.
